# Personals

**Published:** 1918-05

**Authors:** 


### PERSONAL

**Announcement of removal, travel, and other matters of interest are requested. Please report errors in the listing of any physician in the State and other directories, that we may co-operate with the State Society in securing a correct list.**

---

Dr. Marcel Hartwig of Buffalo left April 1 for an extended western and southern trip. Some of his friends surprised him with a dinner and presented him with a traveling bag, having lured him to the Lafeyette Hotel "to see a patient."

Dr. B. F. Illston, Coroner of Chautauqua Co., has been commisioned Captain in the Reserve Corps and left for Ft. Oglethorpe, April 9.

Dr. Lawrence G. Hanley has returned from a trip to Florida.

Dr. George F. Rogan of Medina, was married to Miss Ethel Hughes Beechem, Apr. 3.

